# The Effect of Lavender (*Lavandula stoechas* L.) on Reducing Labor Pain: A Systematic Review and Meta-Analysis

**DOI:** 10.1155/2020/4384350

**Published:** 2020-11-11

**Authors:** Mohsen Kazeminia, Alireza Abdi, Aliakbar Vaisi-Raygani, Rostam Jalali, Shamarina Shohaimi, Alireza Daneshkhah, Nader Salari, Masoud Mohammadi

**Affiliations:** ^1^Department of Nursing, School of Nursing and Midwifery, Kermanshah University of Medical Sciences, Kermanshah, Iran; ^2^Department of Biology, Faculty of Science, University Putra Malaysia, Serdang, Selangor, Malaysia; ^3^School of Computing, Electronics and Maths, Coventry University, London, UK; ^4^Department of Biostatistics, School of Health, Kermanshah University of Medical Sciences, Kermanshah, Iran

## Abstract

**Background:**

Labor pain is one of the most severe pains, which most of women experience. By using novel supportive methods, the labor pain can be reduced, which makes this event pleasant and delightful. Several original studies have been conducted in regard to the effect of lavender on reducing labor pain, whose results are controversial. One of the applications of meta-analysis studies is to respond to these hypotheses and remove controversies; therefore, this study aimed to determine the effect of lavender on labor pain in Iran by using meta-analysis.

**Methods:**

In this study, to find published articles electronically from 2006 to 2019, the published articles in national and international databases of SID, MagIran, IranMedex, IranDoc, Google Scholar, Cochrane Library, Embase, ScienceDirect, Scopus, PubMed, and Web of Science (ISI) were used. Heterogenic index between studies was determined by Cochrane test (Q)c and *I*^2^. Due to heterogeneity, the random effects model was used to estimate standardize difference of the mean score of lavender test in order to assess the labor pain between intervention and control group.

**Results:**

In this meta-analysis and systematic review, finally 13 eligible articles met the inclusion criteria of the study. The sample size from original studies enrolled in the meta-analysis entered in the intervention group was 794 individuals and in the control group was 795 individuals. Mean score for pain in the control group was 7.2 ± 0.42 and in the intervention group was 5.4 ± 0.58 and this difference was statistically significant (*p* ≤ 0.001).

**Conclusion:**

The results of this study showed that lavender can reduce labor pain, which can be considered by health policy makers and gynecologists.

## 1. Background

Although labor is a natural phenomenon, its accompanied pain is severe in more than half of the pregnant women. Most of the women tend to avoid invasive and pharmaceutical methods in management of labor pain, and this issue may increase their tendency to use nonpharmaceutical pain relief methods [1].

Labor pain is one of the most severe pains, which most of women experience. Using novel supportive methods can reduce labor pain and make this event pleasant and delightful [2].

Data from one study showed that perineal massage during the second phase of labor is an appropriate strategy to reduce the number of episiotomy cases and the severity of perineal rupture, possibly due to increased blood flow, elasticity, and perineal softness due to massage [3]. Nonpharmaceutical approach on pain consists of wide spectrum techniques which not only reduce the physical sensation of pain, but also prevent mental suffering resulted from pain care [4].

Aromatherapy using approach is considered for pain today. Controlled use of essence oils for treatment is named aromatherapy. Aromatherapy approach is one of the methods of substitutive medicine in many countries [5], which is applied through various methods such as massage, inhalation, bath, and compress [6]. Massage by midwife results in significant reduction in anxiety and increase in mental support for women in labor [7]. Massage therapy by lavender oil is an effective intervention in reducing the severity of pain in labor that is preferred rather than many nonpharmaceutical methods [8].

Cesarean section is a common surgery around the world, and its prevalence is about 50% of all the labors in Iran [9], and as other surgery procedures, the subsequent pain is reported repeatedly. Controlling of this pain is important for mother in regard to caring for the infant and breastfeeding [10]. Insufficient pain control after cesarean section increases the complications of wounds, infection, and the costs of care process, so that the hospital costs and length of hospital stay are, respectively, 76% and 77% higher than vaginal delivery [11].

Massage is a therapeutic and complementary method applied for various conditions and is of high interest among individuals [8]. Oily essences show the same potency as medications and can be used in a similar way as them, and until more clinical trials are conducted in this field, they should be used by midwives cautiously in the lowest dose and frequency [4]. Studies showed that aromatherapy by using oily essences extracted from aromatic herbs [12] such as lavender, jasmine, and geranium through massage in labor, is effective on mothers' mind [13].

Even when oily essences such as lavender are released in the air and breathed by lungs through uterine contractions, they can produce endorphins in the body to reduce natural pain. In addition, using lavender and *Salvia officinalis* as cold compress on the forehead can help with fatigue and recovery after pain [14].

Lavender, which has long been used in traditional medicine, belongs to family Lamiaceae, and it is also a herbaceous, aromatic, and evergreen herb with a bitter taste and contains volatile oily essence and widely used in aromatherapy [15]. Lavender aroma inhalation decreases anxiety during labor and cortisol secretion and increases serotonin secretion by gastrointestinal tract [16].

In animal studies, lavender oily essence had sedative [17] and analgesic [18] effects. Lavender contains linalool alcohol ketone esterzoaldehyde. Ketones in lavender effectively reduce pain and inflammatory and help falling asleep [18]. Esters prevent muscular spasm, reducing stress and depression [19, 20]. Several original studies in regard to the effect of lavender on reduction in labor pain have been performed, and the results are controversial. One of the applications of meta-analysis studies is clarifying these hypotheses and eliminating controversies. Therefore, this study aimed to determine the effect of lavender on the reduction of labor pain in Iran by using meta-analysis.

## 2. Methods

### 2.1. Literature Review Method

In this study, searching for the Persian databases of SID, MagIran, IranMedex, and IranDoc and international databases of Google Scholar, Cochrane Library, Embase, ScienceDirect, Scopus, PubMed, and Web of Science (ISI) was done in order to find relevant citations from 2006 to 2019. The reference list of all the relevant articles and reports which are found in electronic search was assessed manually to find other probable citations. In case of unavailable articles, a contact was performed by e-mail with authors, and at least a 2-week period was allocated to wait for their response.

### 2.2. Inclusion Criteria

*Criteria for Article Selection*. Articles with the following characteristics were selected for the meta-analysis: (1) original articles, (2) clinical trials (RCT), (3) availability of full texts, and (4) the studies that investigated the association between lavender and reduction of labor pain.

### 2.3. Exclusion Criteria

The articles were performed in review format, or their sample was not selected from the women in labor, and also replicated studies with previous data were excluded.

Used keywords for literature review were selected through Medical Subject Heading (MESH).

Persian keywords were lavender, pain, labor, and cesarean section, and English keywords were lavender, Pain, Giving Birth, Childbirth, Cesarean, Hysterotomy, and Iran.

((((((((((((((lavender[Title/Abstract]) AND (pain[Title/Abstract])) OR (Ache[Title/Abstract])) OR (Suffering[Title/Abstract])) AND (labor pain[Title/Abstract])) OR (Obstetric Pain[Title/Abstract])) OR (Labor, Obstetric[Title/Abstract])) AND (Cesarean Section[Title/Abstract])) OR (Post cesarean Section[Title/Abstract])) OR (Abdominal Delivery[Title/Abstract])) OR (C-Section (OB)[Title/Abstract])) AND (Childbirth[Title/Abstract])) OR (Parturition[Title/Abstract])) OR (Birth[Title/Abstract])) AND (Hysterotomy[Title/Abstract])) OR (Uterus/surgery[Title/Abstract])))))))))))))

In the group of oily essence of lavender, the essence manufactured by Barij Essence Company belonged to *Stoechas* species and was made from unopened flowers through distillation with 1.5% concentration, and its carrier is olive oil; it is guided as cold incense and provided to the mothers by mask. The mothers were asked to inhale through cold incense mask during contraction and pain. Women in the group of massage with lavender oil in each step underwent massage with inhalation of 2 ml lavender oil. For this purpose, the severity of pain was measured by using pain standard assessment scale that is a visual analog scale (VAS) for pain [21]. All the articles had control group (not participated in intervention). Both intervention and control groups were provided with the same standard clinical care.

### 2.4. Qualitative Assessment of the Articles

The quality of articles was assessed based on the selected items and related to CONSORT list which have been pointed out in previous studies. The articles pointing to 6-7 items of criteria were considered as high-quality articles, those pointing to 2 items and those that did not point to more than 2 of 7 items were considered, respectively, as articles with medium-level and low-level methodological quality [22]. In the current study, 13 articles were entered in systematic review and meta-analysis as high- and medium-quality articles, and 6 low-quality articles were omitted (Table 1).

### 2.5. Data Extraction

All the finalized articles entered the meta-analysis were extracted by a preprepared checklist.

The checklist consisted of article title, first author name, publication year, study location, the sample size of intervention and control group, mean sample of intervention and control group, standard deviation of intervention and control group, and the rate of probability.

### 2.6. Statistical Analysis

Since the investigated index was the effect of lavender on labor pain, to combine the results of various studies, frequency and index of standardized mean difference in each study were used. To assess the homogeneity between studies, *I*^2^ index was used, and due to heterogeneity of the studies, random effects model was used in order to combine studies and perform meta-analysis. When the *I*^2^ index is less than 25%, it is known as low heterogeneity, and when *I*^2^ index is between 25 and 75%, it is known as medium heterogeneity and *I*^2^ more than 75% considered as high heterogeneity. *P* value less than 0.05 was considered as significance level. Also, funnel chart and Egger's test were used to assess publication bias.

## 3. Results

**Figure 1:**
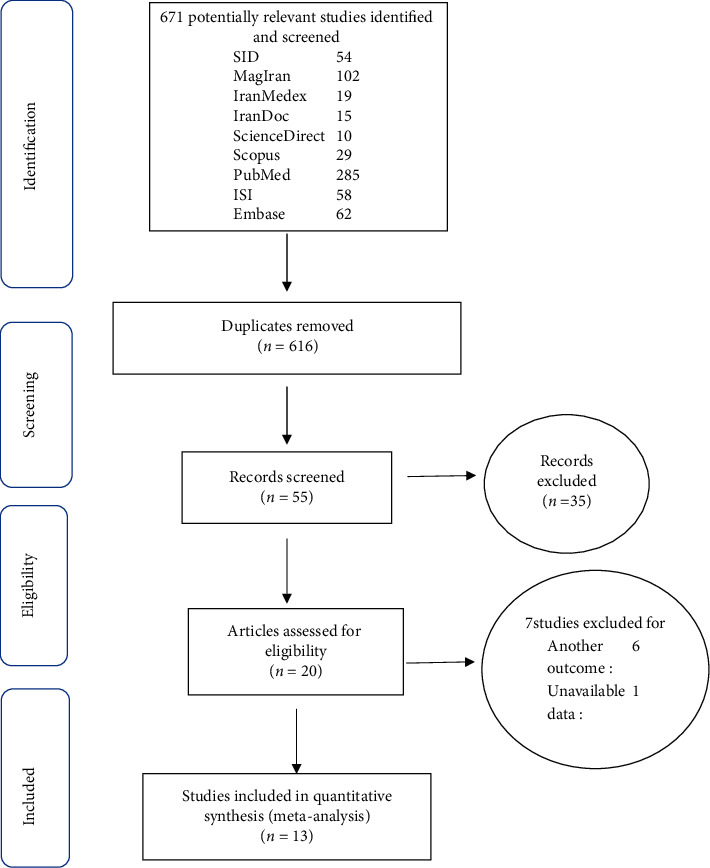
Flow diagram of study selection.

In this study, all the performed researches in regard to the effect of lavender on reduction of labor pain in Iran without time limitation and based on PRISMA guideline were assessed systematically (Figure 1).

In the primary search, 671 articles were identified, and finally 13 articles published between years of 2006 and September 2019 were entered for the final analysis (Table 2). The total sample size was 1589 individuals (795 individuals at control group and 794 individuals at intervention group). The characteristics of entered studies into systematic review were shown in Table 2.

All the studies were clinical trials. Out of 13 articles, 7 articles were published in Persian language and 6 articles were published in English language (Table 2).

**Figure 2:**
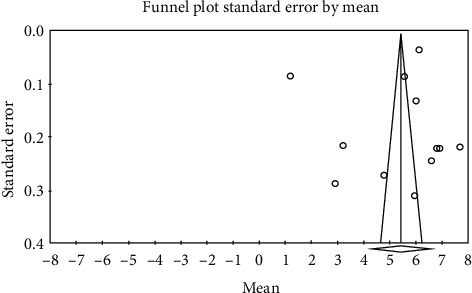
Funnel plot obtained from studies entered into the meta-analysis by using standardized mean difference index (intervention).

**Figure 3:**
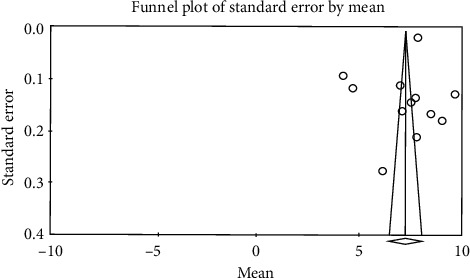
Funnel plot obtained from studies entered into the meta-analysis by using standardized mean difference index (control).

The obtained results from meta-analysis showed that heterogeneity existed between studies; this value for control group was *I*^2^ = 99.5 and for intervention group was *I*^2^ = 99.6; thus, for combining the studies and final estimation of results, random method was used. For determination of publication bias in studies, Egger's test was used. Based on the results from Egger's test, publication bias was not found in studies in intervention group (*p* = 0.733) (Figure 2) and control group (*p* = 0.333) (Figure 3).

**Figure 4:**
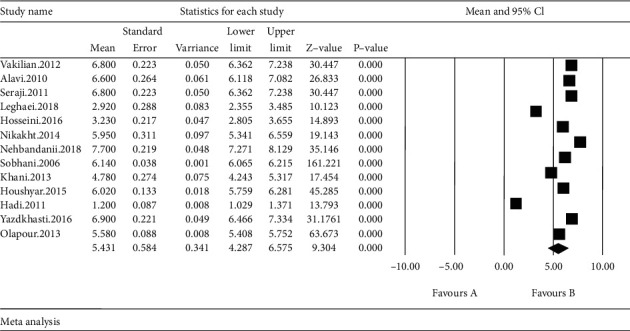
Forest plot obtained by studies entered into the meta-analysis by using standardized mean difference index (intervention).

**Figure 5:**
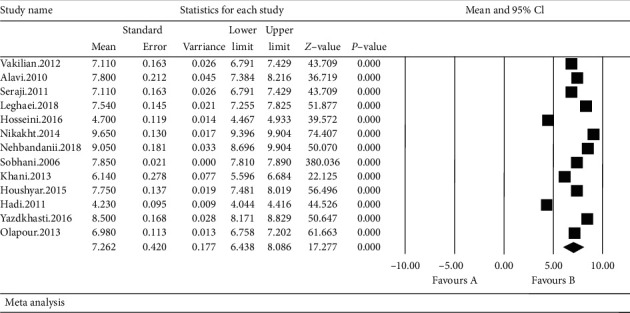
Forest plot obtained by studies entered into the meta-analysis by using standardized mean difference index (control).

The scale obtained in the studies reviewed in the systematic review include the mean and standard error of the Visual Analog Scale; based on the results obtained from meta-analysis, total mean standard error in intervention group was 5.4 ± 0.58 (Figure 4) and total mean standard error in control group was 7.2 ± 0.42 (Figure 5). This difference was statistically significant (*p* ≤ 0.001) which shows that lavender reduces the labor pain. In the forest plot, mean and standard error and confidence interval of 95% in each study and also final estimation of index obtained from combination of studies were shown.

In this chart, weight of each study in final combined value is shown, at which the size of each square is equal to the weight the study implemented in the meta-analysis. Horizontal line of each square shows confidence interval of 95% (Figures 4 and 5).

**Figure 6:**
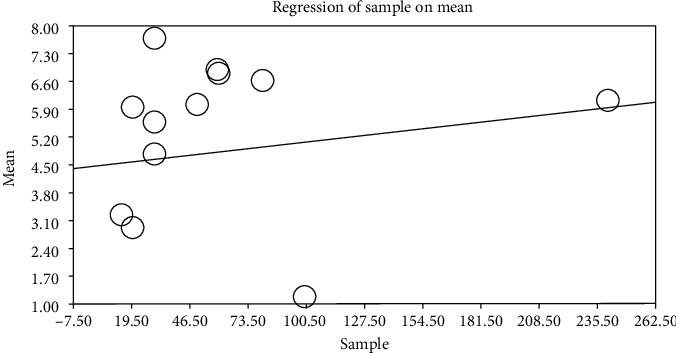
Metaregression of association between sample size and entered studies in meta-analysis by using total mean index (intervention).

**Figure 7:**
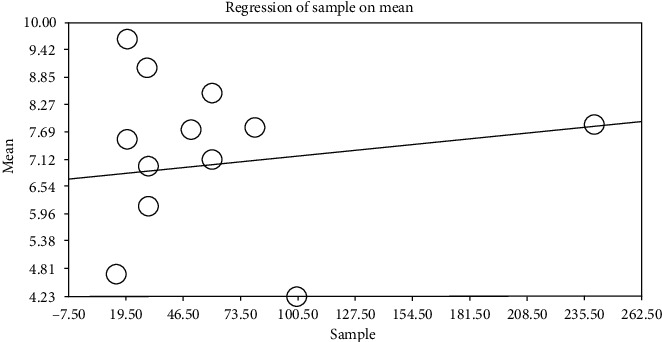
Metaregression of association between sample size and entered studies into meta-analysis by using total mean index (control).

**Figure 8:**
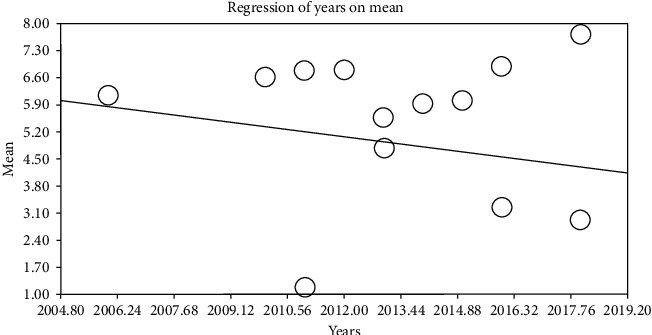
Metaregression of association between publication date and entered studies into the meta-analysis by using total mean index (intervention).

**Figure 9:**
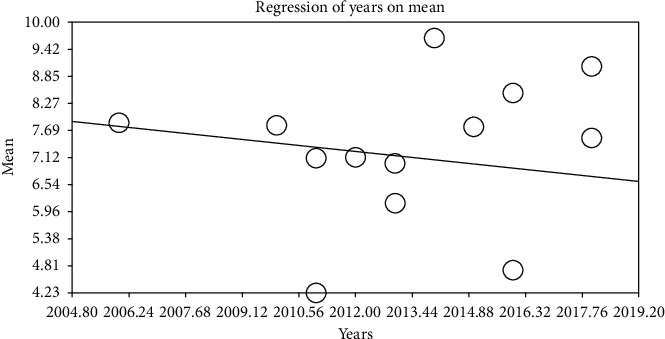
Metaregression of association between publication date and entered studies into the meta-analysis by using total mean index (control).

By using metaregression based on intervention and control groups, the year of performing study (*p* ≤ 0.001) and sample size (*p* ≤ 0.001) (Figures 6–9) with total mean of control and intervention group were assessed, at which there was a significant difference between total mean of intervention and control group; by increasing the sample size in both intervention and control groups, total mean increases (Figures 6 and 7) and by increasing the year of publication in both control and intervention groups, the total mean decreases (Figures 8 and 9).

## 4. Discussion

Since labor is a stressful stage for pregnant individual, catecholamines and cortisol are released in response to pain and anxiety during labor, and by causing severe muscular contraction, this results in uterus muscle hypoxia, and in fact by interfering the labor process, this also causes reduction in energy and increases in mother's fatigue, and by the way it increases the length of labor time [36].

Therefore, this study aimed to determine the effect of lavender on reduction of labor pain in Iran by using meta-analysis. The results of the current study showed a significant difference through investigation of difference between mean scores of labor pain in intervention and control groups.

Estimated total mean in intervention group and control group was 5.4 ± 0.58 and 7.2 ± 0.42, respectively. In confirmation of this finding, Lamadah and Nomani [37] by studying the effect of massage aromatherapy by using lavender oil in regard to the level of pain and anxiety during labor among primiparous women in Egypt (38) and Bronze et al. by studying the effect of aromatherapy on labor outcome in England [38] found results similar to ours.

Studies by Kim et al. investigated the analgesic effects of lavender; the patients underwent breast biopsy by combination of oxygen and lavender supplement 2% which was administered to them by mask, the severity of pain 30 and 60 minutes after the surgery was not less than control group in comparison with patients administered oxygen without lavender, and the requests of patients with lavender group for receiving narcotic analgesic were not less than control group, and therefore the patients showed more satisfaction on pain self-management than control group [39].

In a study using aromatherapy by midwives during labor on 8058 mothers showed that using lavender reduces fear and anxiety of mothers and reduces using epidural anesthesia in this group [38].

The studies showed that lavender aroma is used as a medicinal herb effective in reducing anxiety and pain in mothers during labor, and there is a significant association between reduction in cortisol level and anxiety level [16].

Cortisol is the most important stress hormone [7]. The studies showed that lavender aroma causes reduction in serum cortisol level and subsequently reduction in anxiety and increase in woman's ability for adoption with labor and also reinforcement of narcotic effects and subsequently reduction in narcotic requirement [16].

The probable mechanism of oily essence of lavender is the effect on postsynaptic receptors which is mediated by CAMP and has not any effect on atropine-like receptors [40]. Linalool in lavender causes inhibition of releasing of acetylcholine and changing of ionic channel function in the region of neuromuscular connection and due to that linalyl acetate exhibits narcotic function, and linalool also performs as a sedative; this indicates the use of this herb as a traditional analgesic, and since massage facilitates absorption of volatile oil by skin, linalool and linalyl acetate are rapidly absorbed by skin massage (during 5 minutes), and its plasma concentration reaches the maximum after 19 minutes and disappears in 90 minutes [41, 42].

In aromatherapy, the most important sensations are affected through touch and smell, and while aromatic herbal essences are inhaled, smell impulses are transferred through olfactory receptors to the brain and results in stimulation of limbic system, which subsequently leads to mood moderation, awareness of emotion, maintenance of body temperature, reduction in anxiety and inducing peace emotion, and absorption by skin [43].

According to the obtained results from studies and results from current study, it can be concluded that lavender aroma affects hypothalamus and reduction in secretion of stimulatory hormone of corticotrophin by it through stimulation of olfactory pathways.

Subsequently, the release of adrenocorticotrophin by pituitary gland decreases, and this causes reduction in cortisol secretion by adrenal gland [16, 44].

However, aromatherapy mechanism may be through activation of peripheral neural receptors, which causes reduction in anxiety and fear of mother, and subsequently causes increase in endorphin secretion, pain reduction, catecholamine secretion reduction, and increase of effective uterine contractions induced by reduction in catecholamines and leads to reduction in labor duration [45, 46].

Reduction in labor pain leads to decrease in fatigue and increase in cooperation during labor resulted from the energy reservoir of mother in the second stage of labor and labor process acceleration.

It should be noted that this point is the researcher's perception of scientific texts and its accurate mechanism is not obvious.

One of the limitations in articles was a lack of blinding which is due to the nature of aromatherapy and massage therapy.

Given the results of the current study, to decrease the growing trend of cesarean section which is mainly due to fear of prolongation and the pain of vaginal delivery, aromatherapy and massage with lavender can be helpful, and this method should be involved in educational programs of midwifery and nursing students, and also in the training classes of pregnancy performed for clients and their attendants, this method can be educated to them. Further studies are warranted in regard to safety and quality of specific oils on various patients.

## 5. Conclusion

The high satisfaction resulted from aromatherapy in participants of this study, and the observed significant difference in pain score of intervention group indicates high efficiency of aromatherapy by inhaling lavender essence aroma. Therefore, it is essential to reduce unnecessary cesarean section and treatment costs and promote the health of mothers and infants by making a positive viewpoint through correct announcement to individuals and presentation of positive outcomes by introducing the nonpharmaceutical analgesic methods such as aromatherapy.

## Figures and Tables

**Table 1:** Evaluation of quality of quantitative studies using STROBE tool (*N* = 13).

Row	Author(s) and year of publication	Introduction		Methods							Results							Discussion and conclusion					Score
		Title and abstract	Introduction	Study method	Performing environment	Contributors	Variables	Data source and measurement method	Bias	Sample size	Quantitative variables	Statistical analysis methods	Descriptive data	Participants' reports	Report the original data	Main results of the study	Other analyzes	Report key results	Limitations	Interpretation of results	Generalization	Budget and financial support	
1	Vakilian et al., 2012 [23]	+	+	+	+	+	+	+ +	−	−	+ +	+	+	+	+	+	−	+	+	+	−	−	Medium
2	Alavi et al., 2010 [24]	+	+	+	+	+	+	+ +	−	−	+ +	+	+	+ +	+ +	+	−	+	−	+ +	−	−	High
3	Seraji and Vakilian, 2011 [25]	+	+	+	+	+	+	+ +	−	+	+ +	+	+	+	+	+	+	+	+	+ +	−	−	High
4	Leghaei and Hosseini, 2018 [26]	+	+	+	+	+ +	+ +	+ +	−	+	+ +	+	+	+ +	+	+ +	+	+	+	+ +	−	−	High
5	Hosseini et al., 2016 [27]	+	+	+	+	+	+	+ +	−	+	+ +	+	+	+ +	+	+	+	+	+	+ +	−	−	High
6	Nikbakht et al., 2014 [28]	+	+	+	+	+	+	+ +	−	+	+ +	+	+	+	+	+	+	+	+	+ +	−	−	High
7	Nehbandanii et al., 2018 [29]	+	+	+	+	+	+	+ +	−	+	+ +	+	+	+	+	+	+	+	+	+ +	−	−	High
8	Sobhani et al., 2006 [30]	+	+	+	+	+	+	+ +	−	+	+	+	+	+	+	+	+	+	+	+	−	−	Medium
9	Khani et al., 2013 [31]	+	+	+	+	+	+	+	−	+	+	+	+	+	+	+	+	+	+	+	−	−	Medium
10	Houshyar et al., 2015 [32]	+	+	+	+	+	+	+ +	−	+	+	+	+	+	+	+	+	+	+	+	−	−	Medium
11	Hadi and Hanid, 2011 [33]	+	+	+	+	+	+	+	−	+	+	+	+	+	+	+	+	+	+	+	−	−	Medium
12	Yazdkhasti and Pirak, 2016 [34]	+	+	+	+	+	+	+	−	+	+	+	+	+	+	+	+	+	+	+	−	−	Medium
13	Olapour et al., 2013 [35]	+	+	+	+	+	+	+	−	−	+	+	+	+	+	+	+	+	+	+	−	−	Medium

**Table 2:** Specifications of studies entered into the meta-analysis.

Author, year, reference	Country	Sample size control group	Sample size intervention group	Mean ± SD group intervention	Mean ± SD group control	*P* value
Vakilian, et al., 2012, [23]	Arak	60	60	6.80 ± 1.73	7.11 ± 1.26	0.27
Alavi, et al. 2010, [24]	Shiraz	80	80	6.60 ± 2.20	7.80 ± 1.90	≤0.001
Seraji and Vakilian, 2011, [25]	Arak	60	60	6.85 ± 1.65	7.14 ± 1.30	0.28
Leghaei and Hosseini, 2018, [26]	Shiraz	20	20	2.92 ± 1.29	7.54 ± 0.65	≤0.001
Hosseini, et al., 2016, [27]	Shiraz	15	15	3.23 ± 0.84	4.70 ± 0.46	≤0.001
Nikbakht, et al., 2014, [28]	Mashhad	20	20	5.95 ± 1.39	9.65 ± 0.58	≤0.001
Nehbandanii et al., 2018, [29]	Zabol	30	30	7.70 ± 1.20	9.05 ± 0.99	≤0.001
Sobhani, et al., 2006, [30]	Gilan	240	240	6.14 ± 0.59	7.85 ± 0.32	≤0.001
Khani, et al. 2013, [31]	Birjand	30	30	4.78 ± 1.50	6.14 ± 1.52	≤0.001
Houshyar, et al., 2015, [32]	Kerman	50	50	6.02 ± 0.94	7.75 ± 0.97	≤0.001
Hadi and Hanid, 2011, [33]	Tabriz	100	100	1.20 ± 0.87	4.23 ± 0.95	0.12
Yazdkhasti and Pirak, 2016, [34]	Tehran	60	59	6.90 ± 1.70	8.50 ± 1.30	≤0.001
Olapour, et al., 2013, [35]	Ahvaz	30	30	5.58 ± 0.48	6.98 ± 0.62	≤0.001

## Data Availability

Datasets are available through the corresponding author upon reasonable request.

## Abbreviations

MESH: Medical subject headings
ISI: Web of Science
PRISMA: Preferred Reporting Items for Systematic Reviews and Meta-Analyses.
